# Longitudinal Clinical Features of Post-COVID-19 Patients—Symptoms, Fatigue and Physical Function at 3- and 6-Month Follow-Up

**DOI:** 10.3390/jcm12123966

**Published:** 2023-06-10

**Authors:** Anke Steinmetz, Stefan Gross, Kristin Lehnert, Petra Lücker, Nele Friedrich, Matthias Nauck, Susanne Bahlmann, Jens Fielitz, Marcus Dörr

**Affiliations:** 1 Physical and Rehabilitation Medicine, Department of Trauma, Reconstructive Surgery and Rehabilitation Medicine, University Medicine Greifswald, 17475 Greifswald, Germany; petra.luecker@med.uni-greifswald.de (P.L.); susanne.bahlmann@med.uni-greifswald.de (S.B.); 2 DZHK (German Center for Cardiovascular Research), University Medicine Greifswald, 17475 Greifswald, Germany; stefan.gross1@uni-greifswald.de (S.G.); kristin.lehnert@med.uni-greifswald.de (K.L.); nele.friedrich@med.uni-greifswald.de (N.F.); matthias.nauck@med.uni-greifswald.de (M.N.); 3 Department of Internal Medicine B, University Medicine Greifswald, 17475 Greifswald, Germany; jens.fielitz@med.uni-greifswald.de (J.F.); marcus.doerr@med.uni-greifswald.de (M.D.); 4 Institute of Clinical Chemistry and Laboratory Medicine, University Medicine Greifswald, 17475 Greifswald, Germany

**Keywords:** post-COVID-19-syndrome, long COVID, rehabilitation, physical function, fatigue, psychological profile, 6-min-walk-test, cognitive function, laboratory parameters

## Abstract

Post-COVID-19 syndrome (PCS) has been described as ‘the pandemic after the pandemic’ with more than 65 million people worldwide being affected. The enormous range of symptoms makes both diagnosis complex and treatment difficult. In a post-COVID rehabilitation outpatient clinic, 184 patients, mostly non-hospitalized, received a comprehensive, interdisciplinary diagnostic assessment with fixed follow-up appointments. At baseline, three in four patients reported more than 10 symptoms, the most frequent symptoms were fatigue (84.9%), decreased physical capacity (83.0%), tiredness (81.1%), poor concentration (73.6%), sleeping problems (66.7%) and shortness of breath (67.3%). Abnormalities were found in the mean values of scores for fatigue (FAS = 34.3), cognition (MoCA = 25.5), psychological alterations (anxiety, depression, post-traumatic stress disorder), limitation of lung function (CAT) and severity scores for PCS (PCFS, MCRS). Clinical abnormalities were found in elevated values of heart rate, breathing rate at rest, blood pressure and NT-proBNP levels. As the frequency of the described symptoms decreases only slowly but most often significantly over the course, it is important to monitor the patients over a longer period of time. Many of them suffer from an immense symptom burden, often without pre-existing clinical correlates. Our results show a clear association with objectifiable assessments and tests as well as pronounced symptoms.

## 1. Introduction

More than three years after the onset of the pandemic, it has been shown that COVID-19 can have significant long-term consequences. More than 65 million people worldwide are estimated to be affected by the post-acute COVID-19 syndrome (PCS), commonly differentiated into Long and post-COVID-19-Syndrome [1,2].

The incidence of PCS depends on the definition of the severity of symptoms and varies between 50–70% in hospitalized patients and 10–30% in non-hospitalized patients [3,4]. Vaccinated individuals seem to be affected less frequently, with an incidence of about 10–12% [5,6]. Reinfections with SARS-CoV-2 and increasing age increase the risk of developing PCS [7,8]. The symptoms of PCS show an enormous range with more than 200 different symptoms being described [9,10]. The most frequent symptoms relate to the respiratory and cardiovascular systems, but neurological, psychological and musculoskeletal symptoms are also very common. Fatigue and cognitive deficits are among the greatest impairments. Hence, we are dealing with a complex syndrome usually associated with a combination of a wide variety of symptoms in different organ systems [9]. 

PCS symptoms often persist over a longer period of time. Many patients still suffer from PCS after 6 months to 1 year [11,12,13,14], or even 2 years [15,16], after the infection. This is supported by a meta-analysis, in which a large number of COVID-19 infected individuals still have significant symptoms such as fatigue, dyspnea, sleep disorder and myalgia 9–12 months and even longer than 12 months [17] later. Furthermore, there is a higher risk of adverse outcomes, such as an increased risk for cardiovascular diseases (e.g., cardiac arrhythmia, pulmonary embolism, heart failure) as well as a higher mortality [13].

A lot of studies to date are based on self-reported data or questionnaires without including clinical data or results of examinations. Available clinical data reveal limitations, e.g., in pulmonary function [18,19,20,21]. Improvements in lung function after pulmonary rehabilitation were published by Bouteleux [22]. A significant reduction in terms of muscle strength, flexibility and balance was shown in health care workers with severe COVID-19 infection, which subsequently improved over the course of one year [23]. In addition, reduced walking distance measured by a 6-min walk test (6-MWT) has also been shown [24,25]. Multiorgan impairment assessed by MRI 4 months and 12 months after COVID-19 infection was found by Dennis et al. [14,26]. 

In a meta-analysis, cognitive impairment was found in about every fourth patient with PCS three months or more after the acute infection [27]. There is further evidence for structural and metabolic changes in the brain associated with cognitive impairment following COVID-19 disease [28,29]. Autoantibodies in cerebrospinal fluid were also shown to correlate with cognitive impairment following COVID-19 [30].

So far, there are no specific options to treat PCS. There are still research gaps regarding the onset, the causes of individual symptom distribution and effective therapies. Since the causes of the syndrome are not yet sufficiently understood, symptomatic therapy is often only partially successful. Sometimes, proven therapy options such as physical activity have even negative effects and can seriously worsen patients’ symptoms (e.g., if provided to patients with post-exertional malaise) [31]. Longitudinal studies of the course of the disease under symptomatic therapy will provide an important database for evaluating specific therapeutic approaches and demonstrating a superiority to the standard of care.

A large number of those affected by PCS are working-aged adults, which not only has implications for these individuals, but also places an enormous socioeconomic burden on society and healthcare systems [32]. It is estimated that until November 2022, 31.14 million working-aged adults in the US had developed PCS [33].

In this article, the characteristics, symptoms and follow-up data (at 3 and 6 months) of patients who were cared for in a university post-COVID outpatient clinic are described, and clinical data are correlated with self-reported symptoms and standardized questionnaires.

## 2. Materials and Methods

### 2.1. Study Design

We conducted a prospective cohort study. Inclusion criteria were:

- ≥18 years old
- infection with SARS-CoV-2 (PCR-tested) at least six weeks prior to the initial consultation
- sufficient German language skills.

An acute COVID-19 disease at the time of the initial consultation was an exclusion criterion.

After the initial examination and checking of inclusion criteria, patients were invited to participate in the study. Follow-up appointments were scheduled at 3, 6, 12, 18 and 24 months. Recently, the study design and protocol were published [34]. Here we present the clinical symptoms as well as the outcomes of parts of the assessments. A detailed description of these assessments and methods is provided elsewhere [34]. 

### 2.2. Study Population

**Figure 1:**
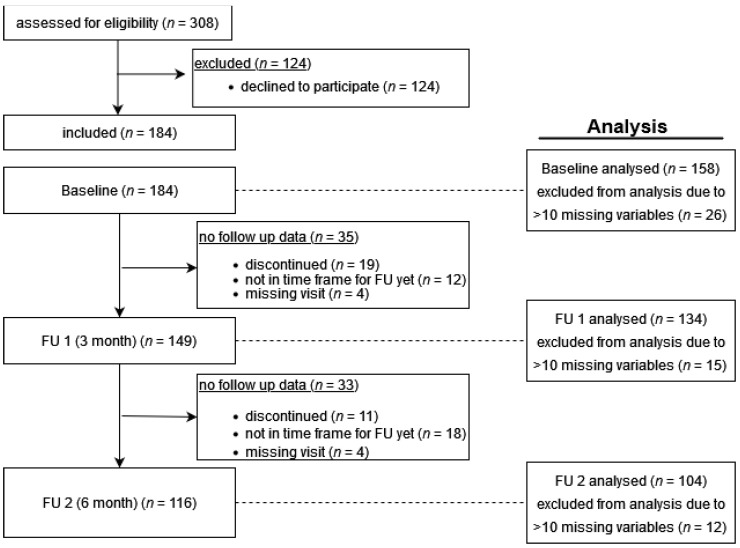
Flowchart–included participants, dropouts, follow-ups.

Patients of a post-COVID- 19 rehabilitation outpatient clinic in Greifswald, Germany, were recruited for this interdisciplinary study, the Greifswald PCS Rehabilitation Study and Research (PoCoRe) [34], since April 2021 (Figure 1). 

Referrals to our specialized outpatient clinic are initiated by primary care physicians in the region of Mecklenburg-Western Pomerania. Therefore, predominantly non-hospitalized patients with a mild acute course of the COVID-19 infection were referred to our university-based clinic. 

### 2.3. Questionnaires

The impairment and symptoms caused by PCS were assessed using various established self-report questionnaires. We here present the Fatigue Assessment Scale (FAS), the Post-COVID-19 Functional Status Scale (PCFS), Veterans Rand 12-Item Health Survey (VR-12), COPD Assessment Test (CAT) and the Median COVID Recovery Score (MCRS), which includes the Generalized Anxiety Disorder Scale-7 (GAD-7), Patient Health Questionnaire-9 (PHQ-9) and International Trauma Questionnaire part 1 (ITQ-1), assessing anxiety, depression and post-traumatic stress disorders. With the exception of the MRCS, these questionnaires are all validated and have been used in other PCS study projects [34]. The questionnaires were completed by the patients during the waiting time before the consultation.

### 2.4. Tests and Examinations

The clinical examination included an orienting physical examination of all organ systems, and measurements of blood pressure (BP), heart rate (HR), respiratory rate (RR) and oxygen saturation (pO_2_). Furthermore, changes in HR, RR and pO_2_ were determined after the 6-MWT, and the patients’ level of exertion was assessed with the Borg Scale. In addition, the Montreal Cognitive Assessment (MoCA) was conducted. Anthropometric parameters were performed at a second appointment at the German Centre for Cardiovascular Research (DZHK). All patients were also offered a psychological consultation.

### 2.5. Laboratory Parameters

Routine parameters included a complete blood count with vitamin D and CRP. In addition, a biosample for subsequent testing was prepared and stored at −80 °C [35]. Baseline parameters ALAT, ASAT, bilirubin, calcium, total cholesterol, creatine kinase, GGT, glucose, HDL cholesterol, LDL cholesterol, uric acid, urea, potassium, creatinine, lipase, sodium, NT-proBNP, phosphate, triglycerides, hs cTroponin and TSH were analysed (Dimension VISTA, Siemens Healthcare Diagnostics, Eschborn, Germany). All assays were performed according to the manufacturers’ recommendations by skilled technical personnel.

### 2.6. Statistics

Statistics was performed with Stata 17.1. (Texas, USA) Statistical analysis included descriptive characteristics of the study population and frequencies of self-reported symptoms with continuous variables given as mean ± SD, categorical variables given as N (%). Differences between males and females were calculated using non-parametric median test or chi-square test. Prevalence of symptoms per visit as well as the presence of symptoms within strata (i.e., either clinically defined categories or tertiles, respectively) of different clinical scores per visit are visualized in a heatmap. Adjusted mean differences between T0, T1 and T2 visits for continuous patient characteristics and continuous clinical scores (see table in Section 3.2) were estimated using linear mixed effects models adjusted for sex, age, diabetes and virus wave when the infection likely occurred. Due to the explorative nature of the present study, we did not adjust for multiple comparisons. Normality of residuals and homoscedasticity were visually checked by QQplots and residuals-vs-fitted plots. We also checked whether those adjusted mean differences between visits were dependent on the virus wave by including the according interaction terms. We included all patients into the analysis who had at least completed the baseline visit T0 (*n* = 158) and had less than ten missing values in their overall total variable set (baseline characteristics/scores/symptoms, *n* = 71 variables considered).

The study protocol was reviewed and approved by the local ethics committee (No. BB 053/21) in accordance with the applicable legal regulations and the Declaration of Helsinki in its current version. Informed consent was obtained from all included patients. The study was prospectively registered in the German Clinical Trials Registry (DRKS 00025007).

## 3. Results

### 3.1. Characteristics of Patients with PCS

Our cohort consisted of 158 patients, included between 20 April 2021 and 1 February 2022, all with PCR testing confirmed COVID-19 infection. A total of 149 individuals were followed up 3 months after the first visit (T1) and 116 patients 6 months after the first visit (T2). The mean age was 48.1 years (SD = 14.2) and the mean body mass index (BMI) 28.8 kg/m^2^ (SD = 6.1). The majority of patients were female (n = 125, 78.6%). On average, the first consultation was 241 days (median 203 days) after COVID-19 infection (range: min. 46 and max. 815 days). All but 22 (18%) patients had not been hospitalized during acute COVID-19 infection which could be classified as mild according to the WHO classification (no oxygen supplementation) [36]. Table 1 shows demographic characteristics for all patients as well as pre-existing comorbidities and allocation to the virus variants based on the date of illness.

### 3.2. Baseline Characteristics and Follow-Up

In general, aerobic capacity and endurance by 6-MWT were impaired on average in all patients at baseline (T0) and improved significantly over follow-ups (T1, T2), see Table 2. Heart rate (HR) at rest and after 6-MWT, respiratory rates (BR), blood pressure (RR) and exertion (measured with the Borg scale) decreased over time. The scores for anxiety and depression also decreased over time, representing a lower grade of impairment. Fatigue and cognition were impaired at baseline and improved at T1 and T2 accordingly. Mean values of MoCA achieved a level of normal function (≥26 pts.) at T1 and improved further to T2 (Table 2). However, in terms of perceived degree of functional improvement, it was found that approximately 57% of participants still had grade 2 (mild functional limitations) at all follow-up visits (Table 3). Nevertheless, the consideration of the severity of the individual scores (Table 3) showed that a substantial number of patients still showed pronounced limitations after 6 months, e.g., 25.0% had an abnormal MoCA score (<26).

### 3.3. Symptoms and Scores over Time 

**Figure 2:**
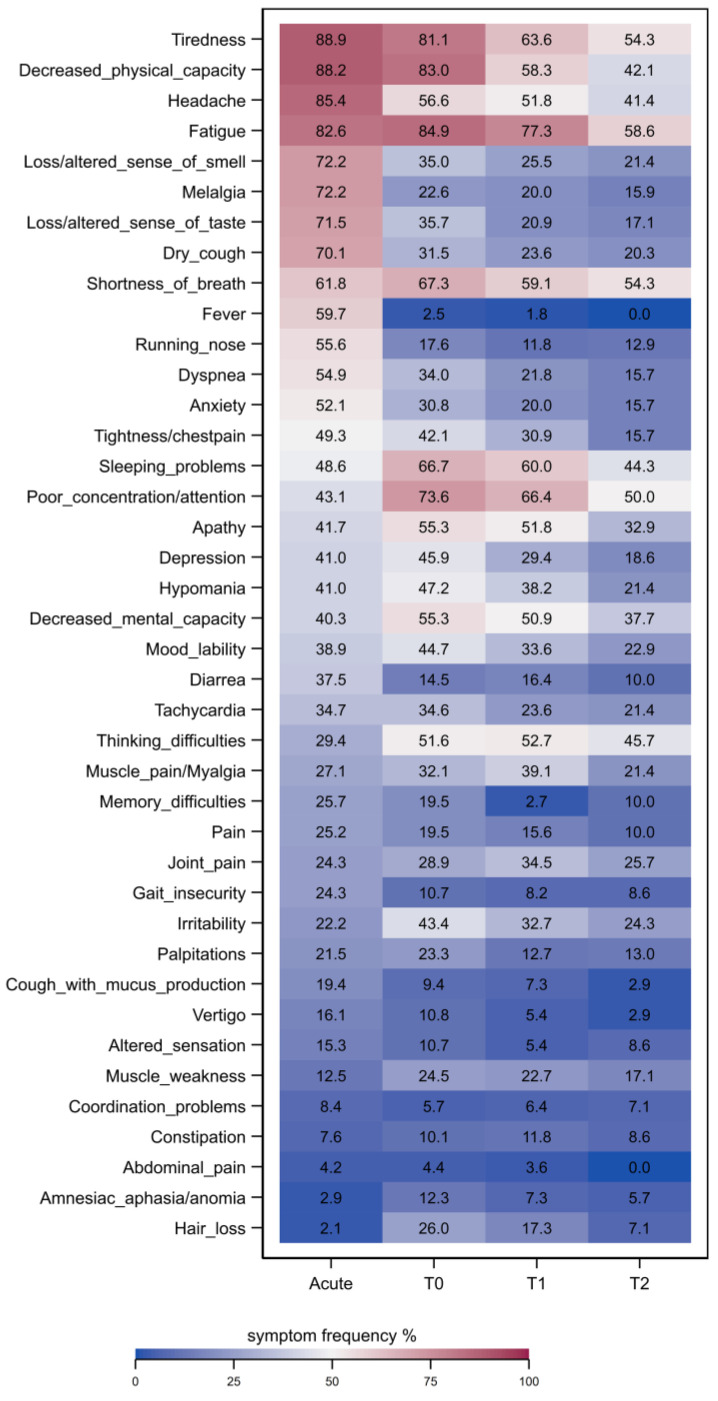
Frequencies of symptoms during acute COVID-19 infection (acute), at baseline (T0), 3-month (T1), and 6-month (T2) follow-up, color intensity depicts frequency (in %) of patients reporting symptoms. Symptoms are ordered from most to least frequent during the acute phase of disease.

Most prevalent (>50%) clinical symptoms in the acute phase of infection were tiredness, decreased physical capacity, headache, fatigue, melalgia, altered sense of smell/taste, a dry cough, shortness of breath, fever, a runny nose, dyspnea and anxiety. For a complete list of clinical symptoms see Figure 2. Clinical symptoms improved in general over time from T0 to T2. However, tiredness, fatigue, shortness of breath and poor concentration/attention were still present in more than 50% of patients at T2. Interestingly, sleeping problems and poor concentration/attention displayed a time delay as two of the most prevalent symptoms during T0 and T1.

The most frequent six symptoms at baseline T0 were fatigue (84.9%), decreased physical capacity (83.0%), tiredness (81.1%), poor concentration/attention (73.6%), sleeping problems (66.7%) and shortness of breath (67.3%) (Figure 2). At T0, three in four patients reported more than 10 of the 31 prespecified symptoms, with an average of 16 symptoms, which decreased to 11 symptoms at T1 and 10 symptoms at T2. 

Detailed complex patterns between frequencies of clinical symptoms and strata of different clinical scores at baseline (T0), T1 and T2 are displayed by heatmaps in Supplementary Figures S1–S3 in the online supplement.

The number of patients unable to work decreased from 47.4% at T0 to 38.2% at T1 and 33.3% at T2.

### 3.4. Laboratory Tests

Laboratory tests did not show particular abnormalities in mean values (Table 4), apart from decreased Vit. D levels, a higher CRP and a slightly higher LDL cholesterol than the normal value. However, 15% of the patients had elevated levels of NT-proBNP. 

## 4. Discussion

We present here the longitudinal course of symptoms, scores and clinical data of patients with PCS from a university post-COVID-19 outpatient clinic over 3 and 6 months. The majority of these patients had COVID-19 in the so-called “2nd Corona Wave” (week 40/20–8/21) and “3rd Corona Wave” (week 9/21–23/21). Thus, the characteristics of PCS presented here relate predominantly to PCS resulting from infections with the SARS-CoV-2 “wild type” (46.9%) and the alpha variant (38.1%). Notably, we present the course of PCS symptoms and limitations over an average period of 8 months after initial infection (241 days) to 6 months thereafter.Since the discussion on the role of psychological factors of PCS seems to be ongoing [37], it is even more important to be able to classify and quantify the symptoms. Although many patients experience an immense symptom burden, there are often only a few objectifiable or pathological findings to be found within the framework of clinical examination parameters. Moreover, other viral diseases (e.g., influenza) also cause relatively non-specific symptoms after the disease [38]. Naturally, PCS symptoms must also be distinguished from pre-existing symptoms, which can be obtained either at the anamnestic level [39] or with electronic health data [4].

Stratification of different symptom clusters or subtypes on the basis of abnormalities in scores or clinical tests is indispensable in order to develop effective therapy options beyond symptomatic treatment. It is also the basis for the differential diagnostic discrimination of PCS from socio-cultural pandemic consequences (e.g., due to the social restrictions) as well as pre-existing or overlapping diseases, especially of psychological origin.

Our patients reported a variety of post-COVID-19 symptoms. On average, 16 symptoms were reported at baseline (IQR 10–19), 74% of the participants reported more than 10 different symptoms. In addition, there were multiple abnormalities in the scores for fatigue (FAS), cognition (MoCA), psychological alterations (anxiety, depression, post-traumatic stress disorder), patients perceived limitation of lung function (CAT) and severity scores for PCS (PCFS, MCRS) were reported. 

At baseline (T1; T2) a total of 90.5% (82.5%; 72.7%) of patients showed fatigue in the FAS, altogether 50% (36.8%; 16.4%) being severe fatigue. Psychological alterations were frequently represented: 43.4% showed a moderate or severe anxiety disorder (21.8%; 18.6%), and 30.2% (18.3%; 9%) a moderate–severe or severe depression at T0 (T1; T2). Furthermore, we also observed signs of a post-traumatic stress disorder represented by elevated ITQ-1 scores. Objective impairment of lung function has been described in various studies throughout the literature [40]. Apart from pulmonary function tests, an evaluation of subjective lung function parameters was performed as well. Patients perceived limitation of lung function with CAT scores of 17.0/40 (14.3/40; 12.1/40) on average, which is considerably higher compared to 10 published in a PCS sample before [41]. 

In addition, clinical abnormalities were found, such as elevated values of heart rate, breathing rate at rest, blood pressure, several laboratory parameters (see Table 3) and the 6-MWT. Particularly interesting was that about 15% of patients had abnormal NT-proBNP levels and thus indications of cardiac insufficiency. 

With regard to reported symptoms, fatigue and tiredness were experienced by 84.9% (81.1%) at baseline, decreasing to 77.3% (63.6%) at T1 and 58.6% (54.3%) at T2. In total, 93.7% of patients with severe fatigue in the FAS at baseline reported fatigue and tiredness. Furthermore, patients with severe fatigue in the FAS score reported sleep problems (75.9%), shortness of breath (77.2%), headache (74.7%), poor attention/concentration difficulties (87.3%), thinking problems (70.9%) and apathy (69.6%). Patients with cognitive impairment in MoCA (<26 pts.) reported concentration/attention difficulties in 83.6% (vs. 67.0% MoCA ≥ 26), memory difficulties 29.5% (vs. 13.4%) and thinking difficulties 62.3% (vs. 45.4%).

Our data show that the frequency of the described symptoms decreased over the course of 6 months (Figure 2). As mentioned, self-reported symptoms correlate with scores from various assessments (e.g., MoCA and thinking problems, poor attention/concentration, FAS and fatigue/tiredness (Figures S1–S3 eSupplement)). 

Over the follow-up time of 6 months, the number of symptoms, scores and test values improved notably, which is reflected in the PCFS, the MCRS and the VR-12. With a reduction in symptom burden, the number of patients who were unable to work decreased from 47.4% at baseline to 38.2% at T1 and 33.3% at 6 months.

### 4.1. Comparison with Other Studies

Fatigue is one of the most common symptoms of PCS patients. In prospective cohort studies following patients with COVID-19, fatigue occurred in 32% of all patients in a meta-analysis [29], the median of this meta-analysis was 2.8 months after infection and the mean was 11.2 months. The meta-analysis by Alkodaymo et al. [17] differentiated the occurrence of symptoms and the time after infection more precisely. They reported that fatigue occurred in 37% of patients 9–12 months after infection and in 41% of patients ≥12 months after the infection. In longitudinal evaluations the frequency of fatigue decreased significantly after 6 months, 12 months or even 2 years [16,25,42]. However, our cohort showed significantly more frequent fatigue symptoms and pronounced fatigue scores, which is probably related to the selection bias of a specialized consultation.

Functional fitness in Chinese health care workers with severe COVID-19 disease was impaired in 70.4% at 5 months, in 48.9% at 8 months and in 29.6% of participants one year after hospital discharge [23]. Impairment of physical fitness in our cohort was reflected by a reduced 6-MWT distance with a mean distance of 460.3 m at baseline, 478.8 m at the 3-month and 499.3 m at the 6-month follow-up. The mean walking distance in a study by González et al. was shorter with 400 m (IQR 362–440 m), whereas Huang et al. reported significantly less impaired 6-MWT distances at 6 months and 2 years, averaging 495 m (440–538 m) and 510 m (453–550), respectively, at 2 years [16,24].

Furthermore, our patients showed increased heart rates and respiratory rates at rest, which increased significantly after the 6MWT despite a rather moderate level of exertion of 13 on average. However, the HR of our patients with an average of 73.5 at rest is quite comparable to the data of Seeßle et al. after 5 months [11]. Ultimately however, the elevated values could also be an indication of ongoing cardiovascular effects of the COVID-19 infection, e.g., in the sense of an affection of the autonomic system. Surprisingly, 15% of participants had elevated NT-proBNP levels, which may also indicate cardiovascular involvement and damage. Elevated levels of this myocardial stress biomarker have already been described in the literature during acute COVID-19 infection and in the post-acute 6–8 weeks [43]. It is remarkable, however, that these changes were still evident in our study 8 months after the acute illness. Whether this is also associated with myocardial dysfunction cannot be clarified at present because the echocardiographic evaluations of our study are not yet available.

Although vitamin D can reduce the risk and severity of acute COVID-19 [44], there are mixed results regarding the role of vitamin D in relation to PCS. Filippo et al. [45] found a significant frequency of reduced vitamin D level in people with PCS than in those without consequences of the disease. However, Townsend et al. found no association of vitamin D levels with persistent fatigue and reduced exercise tolerance, but did not examine other symptoms of PCS in this regard [46]. In this context, it should also be noted that vitamin D levels were usually unknown before the disease.

Cross-sectional or follow-up studies from specialized outpatient clinics are still sparse. Heightman et al. published a study on the 12-month follow-up of 1325 patients with PCS in the first post-COVID-19 clinical service in the UK as early as 2021 [47]. Compared to our cohort, non-hospitalized PCS patients in the UK had fewer comorbidities and were seen earlier within the PCS progress (on average 194 days since symptom onset). Most frequently, patients reported fatigue (63.4%) and breathlessness (60.4%), followed by chest pain (31.1%), myalgia (29.7%), headache (29.3%) and brain fog (24%). All of these symptoms except myalgia occurred more frequently in our cohort. One reason could be the longer time between symptom onset and first consultation, as well as a higher proportion of comorbidities within our cohort. The median of the FAS in our cohort (M = 34) is comparable with one in a study from the UK (M = 30). 

Boesl et al. published the results of the first 100 patients of a specialized neurological outpatient clinic. Cognitive impairment was reported by 72%, of whom 30% scored below 26 points on the MoCA. Fatigue (67%), headache (36%) and persisting hyposmia were also frequently reported and 5.5% of those patients showed signs of severe depression [48]. 

Particularly surprising was that the physical health-related quality of life was found to be dependent on the virus variant. Interestingly, the Omicron variant was associated with the lowest HrQoL at baseline, but increased to the highest HrQoL values at 6-month follow-up. To our knowledge, such a finding has not yet been described in the literature but may also be explained by a selection bias, as patients with infections of the wild and alpha variants may have suffered from post-COVID-19 for significantly longer than those who were infected by the omicron variant more recently.

### 4.2. Strength and Limitations

Some potential limitations need to be discussed regarding the generalizability of our results. As this is a presentation of the clinical course of patients in a special consultation, there is no control data available. As in comparable studies, pre-existing symptoms and co-morbidities were only recorded anamnestically, which can lead to recall bias. Pandemic effects, e.g., due to social restrictions, cannot be ruled out, especially with regard to symptoms such as fatigue, exhaustion and reduced physical and mental resilience. We were, however, able to correlate symptoms to standardized and validated scores and tests, making confounding less likely. Despite this, pandemic- and COVID-19-independent factors (e.g., secondary gain) that play a role in such a long course and contribute to chronification have not been assessed.

The most important issue to consider is potential selection bias. Thus, patients who attend a specialist consultation may be much more affected than patients who consult a general practitioner. Another selection bias is the absence of PCS patients with asymptomatic COVID-19, as one inclusion criterion was proven SARS-CoV-2 infection at the time of illness, so that these patients have not yet been seen in consultation. Although it is not possible to determine exactly which persistent symptoms are exclusively attributable to PCS, our results show a clear association of increased symptom burden with objectifiable assessments and tests.

The presumed selection bias, with possibly more severely ill people with PCS than found in epidemiological studies, certainly limits the generalizability of the data with regard to severity and course of the disease. Nevertheless, our data shows that a majority of patients seeking help in a special consultation are sometimes severely limited and urgently need specific therapies.

Another limitation is that we did not adjust for multiple comparisons (type I error correction) in the present work due to the exploratory and epidemiological observational nature of our analysis when looking for differences between baseline (T0) and follow-up visits T1 and T2. Although this will increase the probability of potentially false null hypothesis rejections (“false positive results”, type I error inflation), it also decreases the chance of overlooking potentially clinically relevant results (type II error deflation). Furthermore, we believe additional confirmatory analyses in follow-up studies as well as validation studies in other post-COVID-19 patient cohorts (with application of type I error corrections) will ensure that the most robust findings of our present study will prevail [49]. 

Finally, we cannot draw any conclusions from this data set regarding the pathomechanisms underlying the reported symptoms. This requires more in-depth analyses, including immunological biomarkers, which we want to carry out in future analyses.

## 5. Conclusions

In summary, non-hospitalized patients with mild acute COVID-19 infection showed a considerable number of sequelae eight months after the initial infection. Basic cardiovascular parameters and endurance levels were significantly abnormal as were standardized psychological screening assessments and tests for fatigue, cognition, anxiety, depression and posttraumatic stress disorders. Patients reported an average of more than 15 symptoms that had persisted since the initial infection, significantly limiting their quality of life and ability to work. 

In conclusion, symptoms and clinical data collected during a specialized post-COVID-19 consultation could be correlated and showed a significant, albeit slow, regression over a follow-up period of 6 months. It is important to recognize that the constellation observed corresponds to that frequently encountered by clinicians and general practitioners in daily practice. Patients are often severely affected, often even after a rather mild acute course of COVID-19, and report many symptoms which can severely limit their daily lives and their ability to work. Although these symptoms slowly resolved with symptomatic therapy, a third of the affected subjects were still not able to work six months after their first consultation in the clinic, i.e., an average of one year after the COVID-19 disease.

Our results illustrate the need for more intensive investigation of symptomatic therapy options and, in particular, the extent to which individual adaptation situation influences the healing process. In clinical practice, patients reporting persistent symptoms after COVID-19 infection should be comprehensively evaluated and examined across all organ systems. As part of future research, Post-COVID should be increasingly investigated in an interdisciplinary manner to allow comprehensive exploration of symptom causes. In addition, efforts to develop specific therapeutic approaches for the various symptoms must be intensified.

## Figures and Tables

**Table 1:** Demographics.

	Female (n = 124)	Male (n = 34)	Total (n = 158)	*p*-Value
Age mean (SD)	48.5 (13.8)	47.1 (16.1)	48.2 (14.3)	0.669
BMI mean (SD)	28.7 (6.2)	29.5 (5.6)	28.9 (6.1)	0.669
Corona Waves n (%)				0.479
1st Wave (incl. summer 2020)	6 (4.8)	1 (2.9)	7 (4.4)	
2nd Wave	54 (43.6)	18 (52.9)	72 (45.6)	
3rd Wave (VOC Alpha) incl. summer 2021	45 (36.3)	8 (23.5)	53 (33.5)	
4th/5th Wave (VOC Delta/Omicron)	19 (15.3)	7 (20.6)	27 (16.5)	
Preexisting comorbidities n (%)				
Diabetes	10 (8.0)	3 (8.8)	13 (8.2)	0.877
Hypertension	32 (25.6)	13 (38.2)	45 (28.5)	0.155
Respiratory diseases *	13 (10.4)	3 (8.2)	16 (10.1)	0.776
Depression	16 (12.9)	3 (8.8)	19 (12.0)	0.517
Rheumatoid arthritis	31 (25.0)	11 (32.3)	42 (26.6)	0.390
Headache	11 (8.9)	1 (2.9)	12 (7.6)	0.248
Smoking status n (%)				
Smoker	14 (13.1)	6 (20.7)	20 (14.7)	0.529
Former smoker	16 (15.0)	3 (10.3)	19 (14.0)	

* Asthma/COPD/pneumonia before COVID-19.

**Table 2:** Clinical Tests and Scores. Mean (SEM) values based on linear mixed effects models adjusted for sex, age, diabetes.

	T0	T1	T2
6-MWT distance	459.8 (6.9)	481.4 (7.4) ***	499.8 (8.3) ***
HR before 6-MWT	80.9 (1.1)	75.2 (1.2) ***	73.5 (1.4) ***
HR after 6-MWT	101.7 (1.6)	97.3 (1.8) *	94.5 (2.1) **
HR change 6-MWT	21.1 (1.5)	22.2 (1.7)	20.8 (2.1)
BR before 6-MWT	16.7 (0.3)	16.1 (0.4)	15.3 (0.4) ***
BR after 6-MWT	22.9 (0.6)	21.2 (0.7) *	20.4 (0.8) **
BR change 6-MWT	6.2 (0.5)	5.3 (0.6)	5.0 (0.7)
Borg Scale 6-MWT (6–20) ^(a)^ p_interaction_ = 0.044			
wk05/20–wk08/21	13.3 (0.3)	12.6 (0.3) **	12.8 (0.3)
wk09/21–wk30/21	12.8 (0.3)	12.2 (0.3) *	11.4 (0.4) ***
wk31/21–wk52/22	14.7 (0.5)	13.9 (0.6)	11.9 (0.9) **
O2 saturation at rest	98.1 (0.2)	97.4 (0.2) **	97.8 (0.2)
O2 saturation after 6-MWT	97.8 (0.1)	97.6 (0.1) *	97.7 (0.1)
BP_sys_	129.4 (1.4)	127.9 (1.5)	123.0 (1.7) ***
BP_dia_	82.0 (1.2)	77.3 (1.4) **	80.1 (1.5)
FAS	34.3 (0.7)	30.9 (0.8) ***	28.2 (0.9) ***
GAD-7	9.1 (0.4)	7.8 (0.4) ***	5.8 (0.5) ***
PHQ-9	11.4 (0.4)	9.4 (0.5) ***	7.9 (0.5) ***
MoCA	25.5 (0.2)	26.2 (0.3) *	26.7 (0.4) **
CAT	17.1 (0.5)	14.3 (0.6) ***	12.1 (0.6) ***
MCRS	14.4 (1.0)	12.6 (1.1)	7.5 (1.4) ***
VR-12 (MCS)	23.5 (1.1)	28.2 (1.2) ***	33.5 (1.3) ***
VR-12 (PCS) ^(a)^ p_interaction_ = 0.007			
wk05/20–wk08/21	33.4 (1.4)	37.8 (1.5) ***	39.4 (1.5) ***
wk09/21–wk30/21	33.2 (1.8)	38.6 (1.8) ***	42.2 (1.9) ***
wk31/21–wk52/22	29.3 (2.6)	30.8 (2.9)	44.9 (3.5) ***
ITQ-1	10.7 (0.7)	9.3 (0.8) *	7.4 (0.9) ***

6-MWT: 6-min walk test, HR: Heart rate; BR: breathing rate, BP_sys/dia_ Blood pressure systolic/diastolic, FAS: Fatigue Assessment Scale, GAD-7: Generalized Anxiety Disorder, PHQ-9: Patient Health Questionnaire-Depression, MoCA: Montreal Cognitive Assessment; CAT: COPD Assessment Test, MRCS: Median COVID Recovery Score, VR-12 (MCS): Veterans Rand 12-item Health Survey-Mental component summary, VR-12 (PCS): Veterans Rand 12-item Health Survey-Physical component summary, ITQ-1: International Trauma Questionnaire-Part I. Statistics: comparisons vs. T0 visit: *: *p* < 0.05, **: *p* < 0.01, ***: *p* < 0.001; ^(a)^ Interaction with virus waves, i.e., differences vs. T0 visit depend on virus wave, in which initial infection occurred. More details on all pair-wise comparisons and exact *p*-values are available in Supplementary Table S1.

**Table 3:** Severity of fatigue/anxiety/depression/MoCA/functional impairment N (%).

	T0	T1	T2
**FAS**			
No fatigue	15 (9.6)	19 (16.7)	21 (27.3)
Fatigue	64 (40.8)	53 (46.5)	39 (50.7)
Severe Fatigue	78 (49.7)	42 (36.8)	17 (16.4)
**GAD-7**			
0 minimal	36 (23.7)	32 (28.8)	42 (53.9)
1 mild	47 (30.9)	44 (39.6)	21 (26.9)
2 moderate	40 (26.3)	21 (18.9)	13 (16.7)
3 severe	29 (19.1)	14 (12.6)	2 (2.6)
**PHQ-9**			
0 minimal	17 (11.1)	22 (20.0)	26 (33.3)
1 mild	48 (31.4)	43 (39.1)	28 (35.9)
2 moderate	43 (28.1)	25 (22.7)	17 (21.8)
3 moderately severe	33 (21.6)	13 (11.8)	7 (9.0)
4 severe	12 (7.8)	7 (6.4)	0 (0.0)
**MoCA**			
<26	61 (38.9)	35 (31.0)	18 (25.0)
>26	96 (61.1)	78 (69.0)	54 (75.0)
**PCFS**			
Grade 0	0 (0.0)	5 (4.4)	10 (13.0)
Grade 1	11 (7.0)	18 (15.8)	16 (20.8)
Grade 2	90 (57.0)	65 (57.0)	44 (57.1)
Grade 3	55 (34.8)	24 (21.0)	7 (9.1)
Grade 4	2 (1.3)	2 (1.8)	0 (0.0)

FAS (Fatigue Assessment Scale) reported as no fatigue (no), fatigue (fatigue) and severe fatigue (severe) according to cutoffs (<21: no, 22–34: fatigue, ≥35 severe fatigue), GAD-7 (Generalized Anxiety Disorder Scale-7) reported as minimal, mild, moderate and severe according to sum score cutoffs (<5: minimal, 5–9: mild, 10–14: moderate, ≥15 severe), PHQ-9 (Patient Health Questionnaire) reported as minimal, mild, moderate, moderately severe and severe symptoms of depression according to sum score (<5: minimal, 5–9: mild, 10–14: moderate, 15–19: moderately, ≥20: severe), MoCA (Montreal Cognitive Assessment) reported below and above cutoff for normal (26 points) (0 = below, 1 above), PCFS (Post-COVID-Functional-Status) reported as scale grade 0, 1, 2, 3 according to patient self-reporting.

**Table 4:** Laboratory tests.

Laboratory Findings	Limits of Normal		T0 Mean (SD)	
Hemoglobin	7.4–10.0/8.6–11.2	mmol/L	8.88 (0.80)	mmol/L
Erythrocytes	4.2–5.4/4.6–6.2	Tpt/L	4.82 (0.41)	Tpt/L
Leukocytes	4.3–10.0	Gpt/L	6.96 1.98)	Gpt/L
Lymphocytes abs.	−5.0	Gpt/L	1.98 (0.61)	Gpt/L
Thrombocytes	140–440	Gpt/L	271.14 (61.82)	Gpt/L
CRP	<5	mg/L	**7.23 (4.14)**	mg/L
Vit. D	>30	µg/L	**29.57 (16.77)**	µg/L
ALAT	<0.77	µkatal/L	0.54 (0.28)	µkatal/L
ASAT	<0.59	µkatal/L	0.36 (0.28)	µkatal/L
Bilirubin	<17	µmol/L	8.41 (4.08)	µmol/L
Calcium	2.12–2.52	mmol/L	2.38 (0.09)	mmol/L
Cholesterol	<6.0	mmol/L	5.72 (1.27)	mmol/L
Creatine kinase	<7.9	µkatal/L	1.75 (0.75)	µkatal/L
GGT	<0.96	µkatal/L	0.52 (1.62)	µkatal/L
Glucose	3.9–6.4	mmol/L	5.66 (0.78)	mmol/L
HDL Cholesterol	>1.03	mmol/L	1.56 (0.46)	mmol/L
Uric acid	155–428	µmol/L	289.00 (93.47)	µmol/L
Urea	2.5–6.4	mmol/L	5.01 (1.18)	mmol/L
Potassium	3.5–4.6	mmol/L	4.02 (0.30)	mmol/L
Creatinine	42–97	µmol/L	67.52 (14.88)	µmol/L
LDL Cholesterol	<3.34	mmol/L	**3.52 (1.10)**	mmol/L
Lipase	1.59–6.36	µkatal/L	2.36 (0.85)	µkatal/L
Sodium	135–145	mmol/L	142.42 (2.48)	mmol/L
NT-proBNP	<125	pg/mL	80.41 (129.82)	pg/mL
Phosphate	0.6–1.6	mmol/L	0.99 (0.16)	mmol/L
Triglycerides	<1.9	mmol/L	1.56 (0.97)	mmol/L
Hs cTroponin I	<59	ng/L	7.46 (5.81)	ng/L
TSH	0.49–3.29	mU/L	1.51 (0.76)	mU/L

## Data Availability

Data of completed analyses will be available from the corresponding author upon reasonable request.

## Supplementary Materials

The following supporting information can be downloaded at: https://www.mdpi.com/article/10.3390/jcm12123966/s1, Figure S1: Frequency (in %) of Symptoms and Scores at baseline (T0); Figure S2: Frequency (in %) of Symptoms and Scores at T1; Figure S3: Frequency (in %) of Symptoms and Scores at T2; Table S1: Detailed change from T0 to T1 to T2.
