# Supplementary material for: Longitudinal Clinical Features of Post-COVID-19 Patients—Symptoms, Fatigue and Physical Function at 3- and 6-Month Follow-Up
Source: J Clin Med. 2023 Jun 10;12(12):3966. doi: 10.3390/jcm12123966 (PMC10299126; doi:10.3390/jcm12123966)
Supplement: Supplementary file 1 [file jcm-12-03966-s001.zip › jcm-2380211-supplementary.pdf]

Supplement

Table S1: Detailed change from T0 to T1 to T2. Statistics: Linear mixed effects model adjusted for corona wave, age, sex and diabetes. [a)]Interaction with virus waves: differences vs T0 visit depend on virus wave, where initial infection occurred.

	T0	T1	T2	T1 vs T0	T2 vs T0	T2 vs T1
**6-MWT distance**	459.8 (6.9)	481.4 (7.4)	499.8 (8.3)	**<0.001**	**<0.001**	**0.011**
**HR before 6-MWT**	80.9 (1.1)	75.2 (1.2)	73.5 (1.4)	**<0.001**	**<0.001**	0.251
**HR after 6-MWT**	101.7 (1.6)	97.3 (1.8)	94.5 (2.1)	**0.020**	**0.001**	0.226
**HR change 6-MWT**	21.1 (1.5)	22.2 (1.7)	20.8 (2.1)	0.573	0.896	0.553
**BR before 6-MWT**	16.7 (0.3)	16.1 (0.4)	15.3 (0.4)	**0.050**	**<0.001**	**<0.001**
**BR after 6-MWT**	22.9 (0.6)	21.2 (0.7)	20.4 (0.8)	**0.017**	**0.002**	0.302
**BR change 6-MWT**	6.2 (0.5)	5.3 (0.6)	5.0 (0.7)	0.208	0.123	0.667
**Borg Scale 6-MWT (6–20)[a)]**						
$p_{interaction}$ **= 0.044**						
**wk05/20–wk08/21**	13.3 (0.3)	12.6 (0.3)	12.8 (0.3)	**<0.001**	**<0.001**	**<0.001**
**wk09/21–wk30/21**	12.8 (0.3)	12.2 (0.3)	11.4 (0.4)	**0.043**	**<0.001**	**0.044**
**wk31/21–wk52/22**	14.7 (0.5)	13.9 (0.6)	11.9 (0.9)	0.161	**0.001**	**0.026**
**O2 saturation at rest**	98.1 (0.2)	97.4 (0.2)	97.8 (0.2)	**0.002**	0.210	0.148
**O2 saturation after 6-MWT**	97.8 (0.1)	97.6 (0.1)	97.7 (0.1)	**0.027**	0.208	0.534
BP_{sys}	129.4 (1.4)	127.9 (1.5)	123.0 (1.7)			
BP_{dia}	82.0 (1.2)	77.3 (1.4)	80.1 (1.5)			
**FAS**	34.3 (0.7)	30.9 (0.8)	28.2 (0.9)	**<0.001**	**<0.001**	**0.003**
**GAD-7**	9.1 (0.4)	7.8 (0.4)	5.8 (0.5)	**<0.001**	**<0.001**	**<0.001**
**PHQ-9**	11.4 (0.4)	9.4 (0.5)	7.9 (0.5)	**<0.001**	**<0.001**	**0.002**
**MoCA**	25.5 (0.2)	26.2 (0.3)	26.7 (0.4)	**0.016**	**<0.001**	0.125
**CAT**	17.1 (0.5)	14.3 (0.6)	12.1 (0.6)	**<0.001**	**<0.001**	**<0.001**
**MCRS**	14.4 (1.0)	12.6 (1.1)	7.5 (1.4)	0.190	**<0.001**	**0.003**
**VR-12 (MCS)**	23.5 (1.1)	28.2 (1.2)	33.5 (1.3)	**<0.001**	**<0.001**	**<0.001**
**VR-12 (PCS)[a)]**						
$p_{interaction}$ **= 0.007**						
**wk05/20–wk08/21**	33.4 (1.4)	37.8 (1.5)	39.4 (1.5)	**<0.001**	**<0.001**	0.187
**wk09/21–wk30/21**	33.2 (1.8)	38.6 (1.8)	42.2 (1.9)	**<0.001**	**<0.001**	**<0.001**
**wk31/21–wk52/22**	29.3 (2.6)	30.8 (2.9)	44.9 (3.5)	0.536	**<0.001**	**<0.001**
**ITQ-1**	10.7 (0.7)	9.3 (0.8)	7.4 (0.9)	**0.040**	**<0.001**	**0.014**

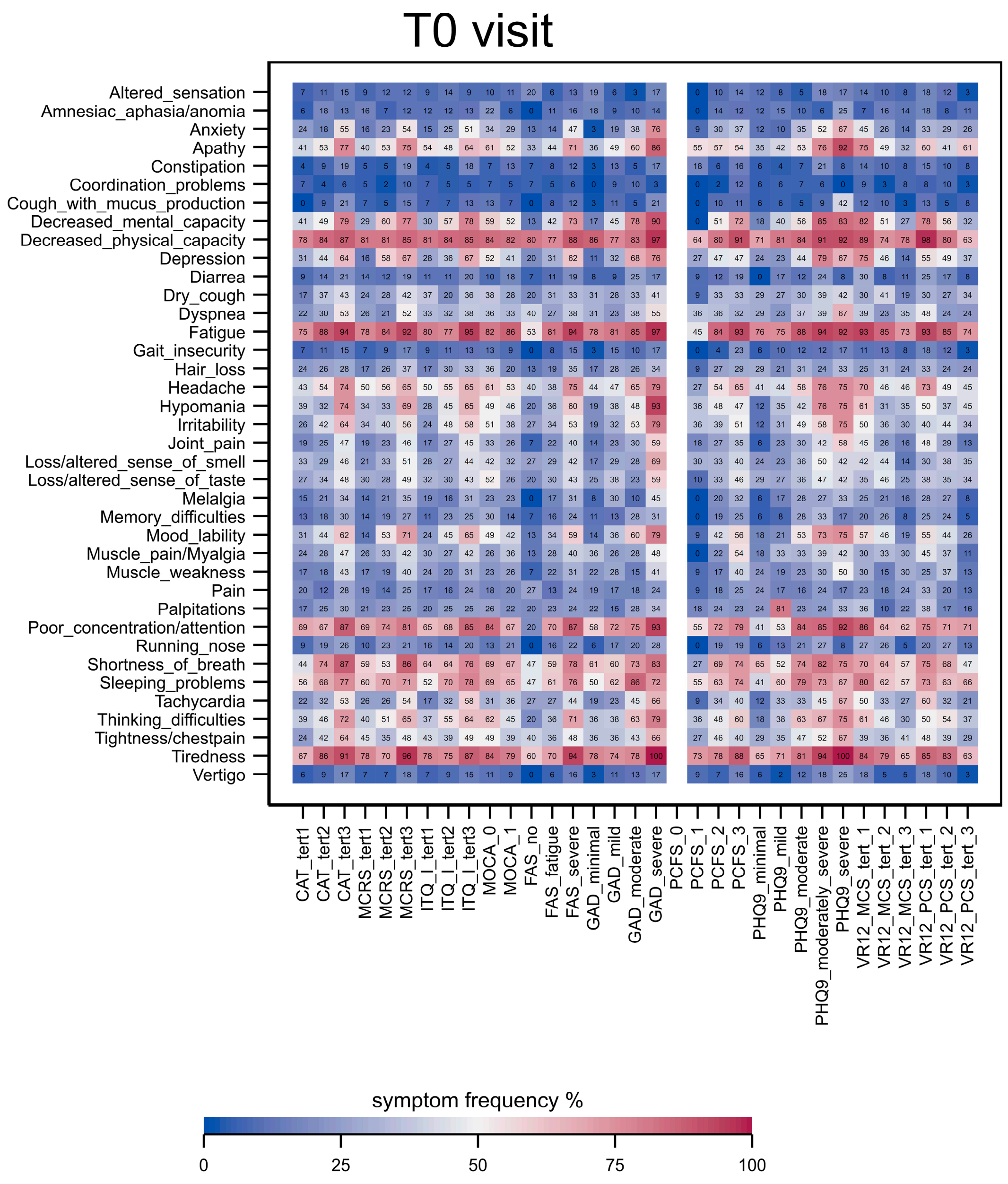

Figure S1: Frequency (in %) of Symptoms and Scores at baseline (T0)

CAT (COPD Assessment Test) , MCRS (Median COVID Recovery Score) ITQ-I (International Trauma Questionnaire part 1), and VR12 (Veterans Rand 12-Item Health Survey) reported in tertiles of sample distribution (tert1, tert2, tert3); MoCA (Montreal Cognitive Assessment) reported below and above cutoff for normal (26 points) (0=below, 1 above), FAS (Fatigue Assessment Scale) reported as no fatigue (no), fatigue (fatigue) and severe fatigue (severe) according to cutoffs (<21: no, 22-34: fatigue, ≥35severe fatigue), GAD-7 (Generalized Anxiety Disorder Scale-7) reported as minimal, mild, moderate and severe according to sum score cutoffs (<5: minimal, 5-9: mild, 10-14: moderate, ≥15 severe), PCFS (Post-COVID-Functional-Status) reported as scale grade 0, 1, 2, 3 according to patient self report, PHQ-9 (Patient Health Questionnaire) reported as minimal, mild, moderate moderately severe and severe symptoms of depression according to sum score (<5: minimal, 5-9: mild, 10-14: moderate, 15-19: moderately, ≥ 20: severe)

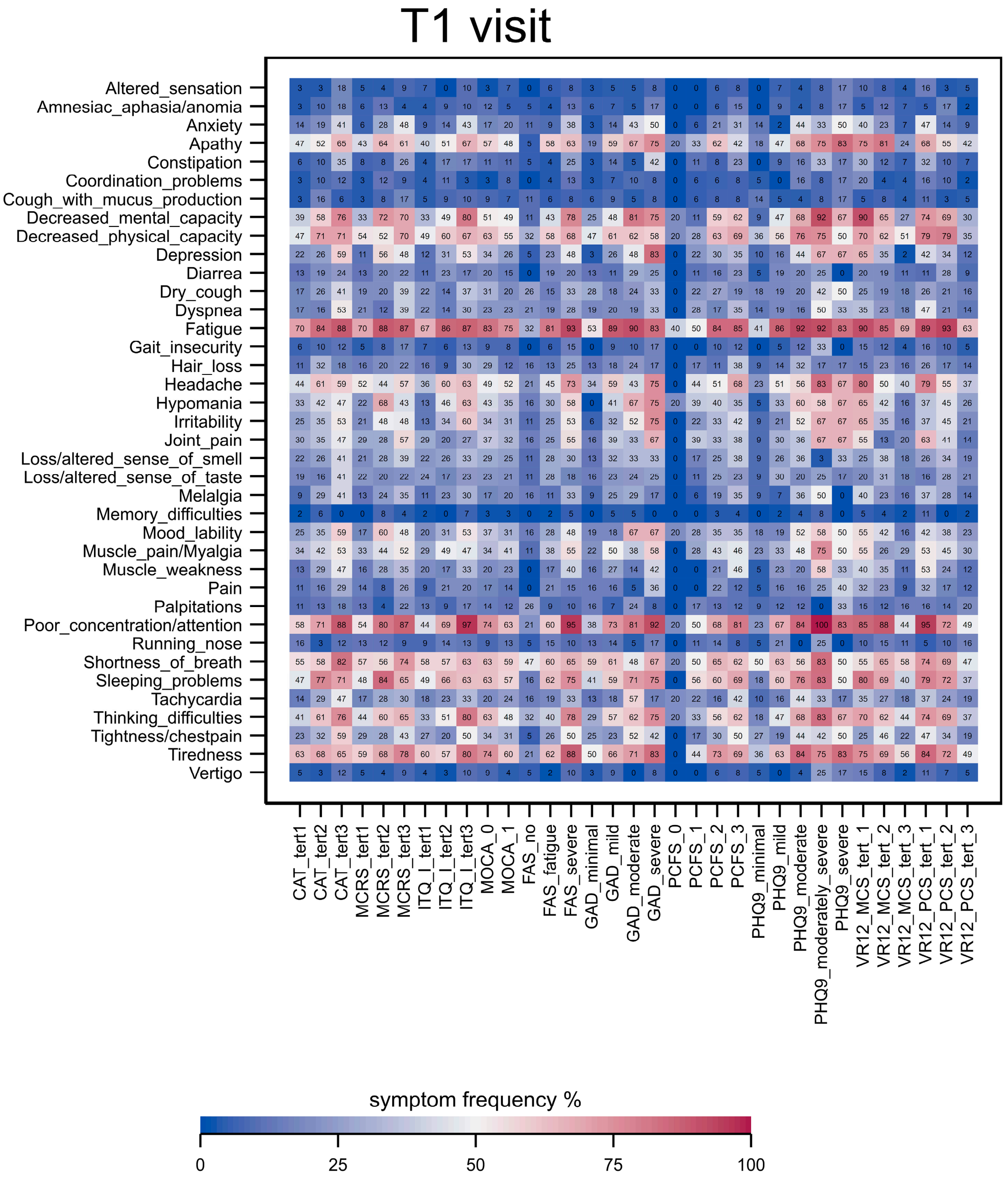

Figure S2: Frequency (in %) of Symptoms and Scores at T1

CAT (COPD Assessment Test) , MCRS (Median COVID Recovery Score) ITQ-I (Posttraumatic Stress Disorder), and VR12 (Veterans Rand 12-Item Health Survey) reported in tertiles of sample distribution (tert1, tert2, tert3); MoCA (Montreal Cognitive Assessment) reported below and above cutoff for normal (26 points) (0=below, 1 above), FAS (Fatigue Assessment Scale) reported as no fatigue (no), fatigue (fatigue) and severe fatigue (severe) according to cutoffs (<21: no, 22-34: fatigue, ≥35severe fatigue), GAD-7 (Generalized Anxiety Disorder Scale-7) reported as minimal, mild, moderate and severe according to sum score cutoffs (<5: minimal, 5-9: mild, 10-14: moderate, ≥15 severe), PCFS (Post-COVID-Functional-Status) reported as scale grade 0, 1, 2, 3 according to patient self report, PHQ-9 (Patient Health Questionnaire) reported as minimal, mild, moderate moderately severe and severe symptoms of depression according to sum score (<5: minimal, 5-9: mild, 10-14: moderate, 15-19: moderately, ≥ 20: severe)

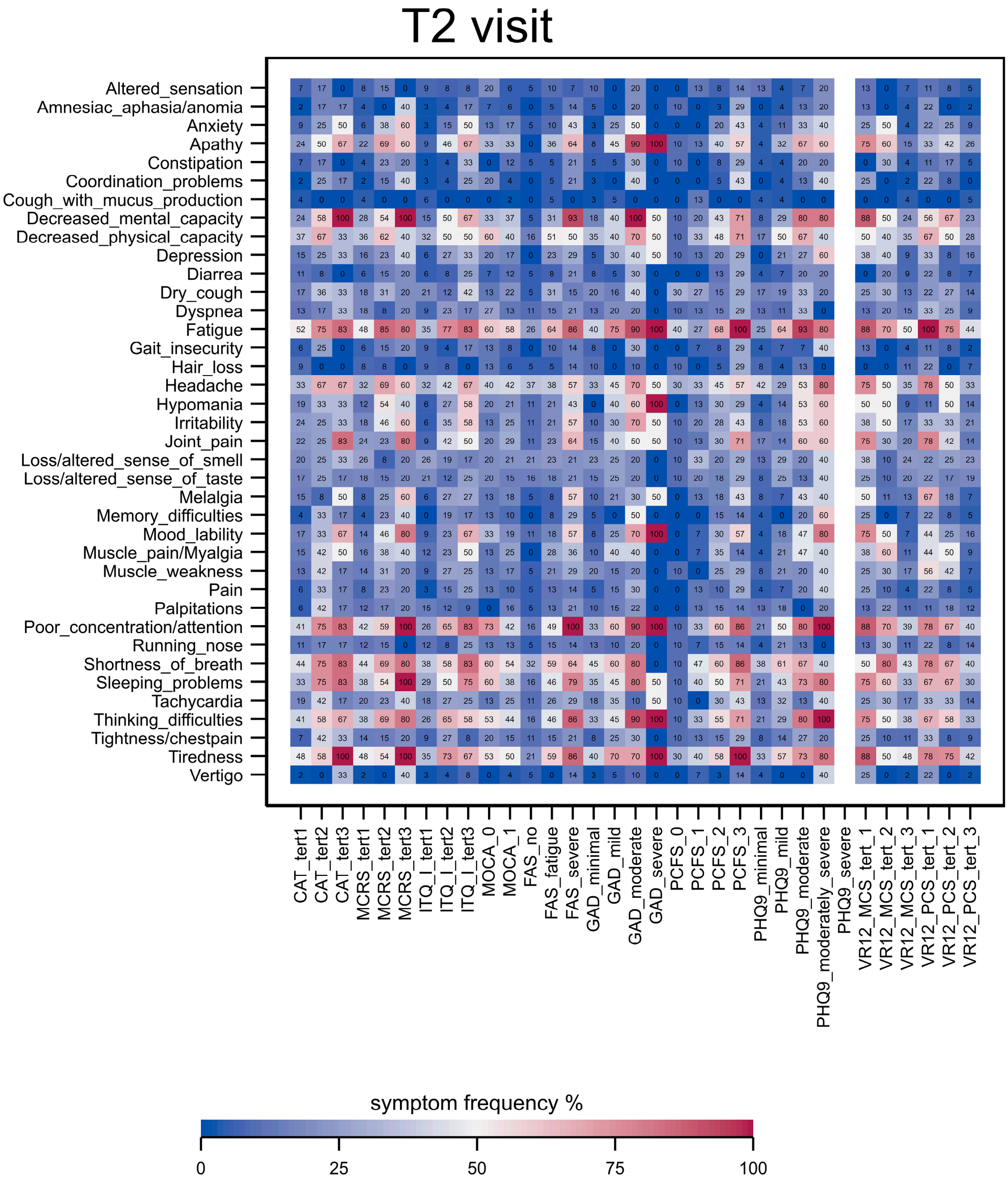

Figure S3: Frequency (in %) of Symptoms and Scores at T2

CAT (COPD Assessment Test) , MCRS (Median COVID Recovery Score) ITQ-I (Posttraumatic Stress Disorder), and VR12 (Veterans Rand 12-Item Health Survey) reported in tertiles of sample distribution (tert1, tert2, tert3); MoCA (Montreal Cognitive Assessment) reported below and above cutoff for normal (26 points) (0=below, 1 above), FAS (Fatigue Assessment Scale) reported as no fatigue (no), fatigue (fatigue) and severe fatigue (severe) according to cutoffs (<21: no, 22-34: fatigue, ≥35severe fatigue), GAD-7 (Generalized Anxiety Disorder Scale-7) reported as minimal, mild, moderate and severe according to sum score cutoffs (<5: minimal, 5-9: mild, 10-14: moderate, ≥15 severe), PCFS (Post-COVID-Functional-Status) reported as scale grade 0, 1, 2, 3 according to patient self report, PHQ-9 (Patient Health Questionnaire) reported as minimal, mild, moderate moderately severe and severe symptoms of depression according to sum score (<5: minimal, 5-9: mild, 10-14: moderate, 15-19: moderately, ≥ 20: severe)
